# Varicella‐Zoster Virus‐Associated Esophagogastritis

**DOI:** 10.1155/crgm/9319708

**Published:** 2026-04-15

**Authors:** Masaya Iwamuro, Takehiro Tanaka, Motoyuki Otsuka

**Affiliations:** ^1^ Department of Gastroenterology and Hepatology, Graduate School of Medicine, Dentistry, and Pharmaceutical Sciences, Okayama University, Okayama, 700-8558, Japan, okayama-u.ac.jp; ^2^ Department of Pathology, Graduate School of Medicine, Dentistry, and Pharmaceutical Sciences, Okayama University, Okayama, 700-8558, Japan, okayama-u.ac.jp

**Keywords:** chemotherapy, endoscopic biopsy, varicella-zoster virus

## Abstract

Varicella‐zoster virus (VZV) typically manifests as varicella or herpes zoster; however, in immunocompromised patients, viral reactivation may result in disseminated infection with visceral organ involvement. Gastrointestinal involvement is particularly rare and often underrecognized. We report a case of biopsy‐proven VZV‐associated esophagogastritis in a patient with Richter syndrome undergoing chemotherapy. A 77‐year‐old man was admitted with abdominal pain, anorexia, and fatigue. On hospital Day 3, he developed disseminated papulovesicular skin lesions. Cytologic examination of vesicular fluid revealed multinucleated giant cells, and antigen testing confirmed VZV infection. Esophagogastroduodenoscopy demonstrated erosions with white exudates in the esophagus and multiple gastric ulcers. Biopsy specimens from both the esophagus and stomach showed positive immunohistochemical staining for VZV. In addition, esophageal specimens exhibited characteristic viral cytopathic changes, including intranuclear inclusion bodies and multinucleation, confirming the diagnosis of VZV‐associated esophagogastritis. Intravenous acyclovir therapy resulted in marked improvement of both cutaneous and gastrointestinal lesions. VZV‐associated esophagogastritis is a rare but clinically significant manifestation of disseminated VZV infection. This condition should be considered in immunocompromised patients presenting with unexplained gastrointestinal symptoms, even before the appearance of characteristic skin lesions. Early endoscopic evaluation with biopsy and appropriate virological testing is essential for timely diagnosis and effective antiviral treatment.

## 1. Introduction

Varicella‐zoster virus (VZV) typically causes varicella as a primary infection and herpes zoster upon reactivation. In immunocompromised patients, however, viral reactivation may result in disseminated infection with visceral organ involvement rather than isolated cutaneous disease [1–4].

Gastrointestinal involvement of VZV is particularly rare and remains poorly recognized. A review of the literature reveals that only nine cases have demonstrated VZV involvement of gastric lesions confirmed by immunohistochemistry or polymerase chain reaction, highlighting the extreme rarity of biopsy‐proven VZV gastritis. Clinical recognition is further complicated by the fact that cutaneous manifestations may develop after the onset of gastrointestinal symptoms or may be absent at presentation, which can delay consideration of VZV infection [5–7].

## 2. Case Presentation

A 77‐year‐old Japanese man with Richter syndrome—transformation of chronic lymphocytic leukemia into diffuse large B‐cell lymphoma—was urgently admitted because of abdominal pain, loss of appetite, and general fatigue that had developed two days prior to admission. No skin eruptions were observed at the time of admission. The patient had no known history of varicella or herpes zoster. He had previously undergone six cycles of chemotherapy consisting of rituximab, cyclophosphamide, doxorubicin, vincristine, and prednisolone, although residual disease persisted at the time of admission.

Abdominal pain, loss of appetite, and general fatigue slightly improved after admission but persisted. On hospital Day 3, he developed scattered papules over the face, trunk, and extremities, some of which evolved into erythematous and vesicular eruptions with halos. Cytologic examination of vesicular fluid demonstrated multinucleated giant cells, and antigen testing was positive for VZV, supporting the diagnosis of disseminated cutaneous VZV infection. Laboratory tests (Table 1) showed a white blood cell count of 1450/μL, with neutrophils 50% and lymphocytes 31.5%; the platelet count was reduced to 29,000/μL. Serum total protein was 5.2 g/dL and albumin 3.5 g/dL, both decreased. Liver and biliary enzymes were elevated: aspartate aminotransferase 436 U/L, alanine aminotransferase 457 U/L, alkaline phosphatase 237 U/L, gamma‐glutamyl transpeptidase 570 U/L, and lactate dehydrogenase 565 U/L. Blood urea nitrogen was elevated at 27.7 mg/dL, and amylase at 199 U/L. C‐reactive protein was slightly elevated at 0.93 mg/dL.

**TABLE 1:** Laboratory findings.

Blood test results (units)	Patient value	Reference range
White blood cells (/μL)	1450	3300–8600
Neutrophil (%)	50	40–70
Lymphocyte (%)	31.5	16.5–49.5
Monocyte (%)	18	2–10
Red blood cells (/μL)	4.66 × 10^6^	4.35 × 10^6^–5.55 × 10^6^
Hemoglobin (g/dL)	14.5	13.7–16.8
Hematocrit (%)	44.8	40.7–50.1
Platelets (/μL)	29 × 10^3^	158 × 10^3^–348 × 10^3^
Total protein (g/dL)	5.2	6.6–8.1
Albumin (g/dL)	3.5	4.1–5.1
Creatinine (mg/dL)	0.93	0.65–1.07
Sodium (mmol/L)	137	138–145
Potassium (mmol/L)	4	3.6–4.8
Total bilirubin (mg/dL)	0.98	0.4–1.5
Conjugated bilirubin (mg/dL)	0.23	0.0–0.2
Aspartate aminotransferase (U/L)	436	13–30
Alanine aminotransferase (U/L)	457	10–42
γ‐Glutamyl transpeptidase (U/L)	570	38–113
Lactate dehydrogenase (U/L)	565	124–222
Alkaline phosphatase (U/L)	237	38–113
Amylase (U/L)	199	44–132
C‐reactive protein (mg/dL)	0.93	0.02–0.15
D‐dimer (μg/mL)	2.7	0–0.9
Prothrombin time (%)	124	73–118
Activated partial thromboplastin time (s)	26.1	24–34

**FIGURE 1:**
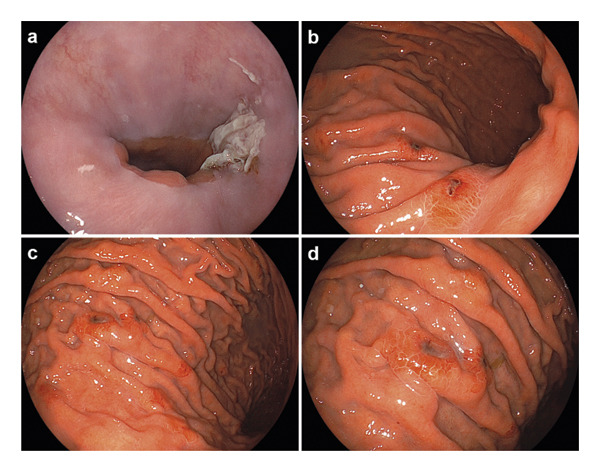
Endoscopic findings of varicella‐zoster virus (VZV)–induced esophagogastritis. (a) Esophagogastroduodenoscopy showing erosive esophageal mucosa covered with white exudates. (b–d) Multiple gastric ulcers with surrounding erythema observed in the gastric body.

**FIGURE 2:**
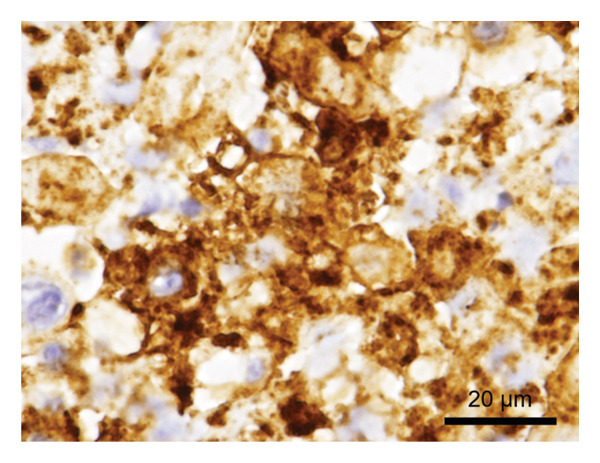
Immunohistochemical findings of VZV infection. Immunohistochemical staining of biopsy specimens from the esophagus and stomach demonstrating positive staining for VZV antigen (scale bar = 20 μm).

**FIGURE 3:**
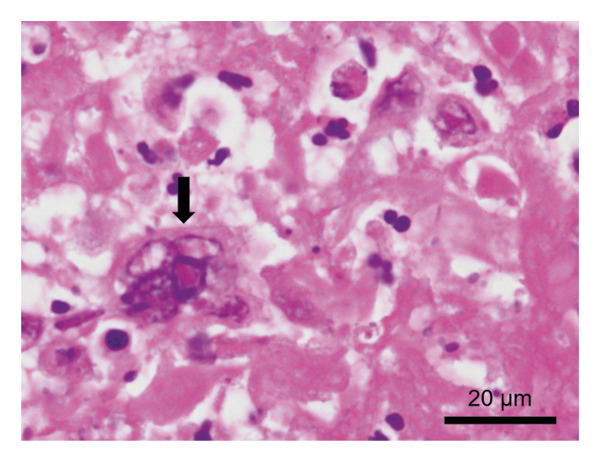
Histopathological findings of esophageal biopsy specimens. Hematoxylin and eosin staining showing epithelial cells with intranuclear inclusion bodies and multinucleation (arrow), findings characteristic of VZV infection (scale bar = 20 μm).

Because his gastrointestinal symptoms persisted, esophagogastroduodenoscopy was performed on the same day. It revealed patchy erosions covered with white exudates in the esophagus (Figure 1(a)), and multiple gastric ulcers surrounded by erythema (Figures 1(b), 1(c), and 1(d)). Targeted biopsies were obtained from both lesions. Immunohistochemical staining for VZV antigen was positive in the esophageal and gastric specimens (Figure 2). Esophageal tissue additionally demonstrated intranuclear inclusion bodies and multinucleation within epithelial cells, confirming VZV‐induced esophagogastritis (Figure 3).

A diagnosis of visceral disseminated VZV infection was established. Intravenous acyclovir therapy (5 mg/kg every 8 h) was initiated and continued for 14 days, which resulted in gradual resolution of the cutaneous lesions. Follow‐up esophagogastroduodenoscopy performed 28 days after the initiation of treatment demonstrated significant improvement of the esophageal and gastric abnormalities.

## 3. Discussion

In the present case, the diagnostic process was particularly challenging during the early phase of hospitalization. Before the appearance of cutaneous lesions, the differential diagnosis for his persistent abdominal pain and anorexia included chemotherapy‐related mucosal injury, neutropenic enterocolitis, cytomegalovirus infection, herpes simplex virus infection, Candida infection, peptic ulcer disease, drug‐induced gastritis, and possible gastrointestinal involvement of lymphoma. Given his profound immunosuppression and cytopenia, opportunistic infections were strongly considered. However, in the absence of characteristic skin eruptions at that time, VZV infection was not initially suspected.

In immunocompromised patients, infectious esophagitis due to herpes simplex virus and cytomegalovirus should be considered when evaluating esophageal erosions or ulcers. Herpes simplex virus esophagitis typically involves the mid‐to‐distal esophagus and presents endoscopically as multiple small ulcers, often with raised margins; more extensive disease may appear as confluent, map‐like ulcers [8]. In contrast, cytomegalovirus esophagitis more commonly manifests as large, deep, linear, or longitudinal ulcers. Candida esophagitis is characterized by adherent white plaques or pseudomembranes. Because the endoscopic appearance of VZV esophagitis is heterogeneous and may overlap with these entities, histopathologic evaluation with virological confirmation (immunohistochemistry or polymerase chain reaction) is essential for accurate diagnosis.

Visceral gastrointestinal involvement of VZV is an uncommon but clinically important manifestation of disseminated infection, occurring predominantly in immunocompromised patients, including those with hematologic malignancies, recipients of chemotherapy, and transplant recipients [5–7, 9–11]. Our patient, who had Richter syndrome under active chemotherapy, represents a population at particularly high risk for severe viral reactivation due to profound disease‐ and treatment‐related immunosuppression.

To clarify the rarity of this condition, we conducted a literature search in the PubMed database using the keywords “varicella” and “gastritis.” Only nine published cases were identified in which VZV involvement of gastric lesions was confirmed by immunohistochemistry or polymerase chain reaction (Table 2) [6, 12–19]. This limited number of confirmed cases suggests that VZV gastritis is rare and may be underdiagnosed in clinical practice.

**TABLE 2:** Reported cases of biopsy‐proven varicella‐zoster virus–associated gastritis.

Author	Year	Age	Sex	Manifestation	Underlying diseases	Endoscopic features of the gastric lesions	Rash onset	Diagnosis of VZV gastritis	Treatment
McCluggage WG	1994	33	M	Abdominal pain with vomiting	Hodgkin lymphoma, postautologous stem cell transplantation	Patchy gastritis with multiple superficial erosions	After abdominal symptoms	PCR	Intravenous acyclovir
Rivera‐Vaquerizo PA	2001	41	F	Abdominal pain with vomiting and nausea	Acute promyelocytic leukemia, postchemotherapy, and stem cell transplantation	Elevated congestive lesions	After 72 h of admission	PCR	Intravenous acyclovir
Stratman E	2002	77	M	Thoracic pain with vomiting and anorexia	Non‐Hodgkin lymphoma, postchemotherapy	Erosive gastritis with multiple shallow ulcers	Present	IHC	Intravenous acyclovir followed by oral valacyclovir
Kim DG	2012	42	F	Abdominal pain with anorexia	None	Multiple erosive lesions with slightly raised erythematous margins	7 days before presentation	PCR	Intravenous acyclovir
Ugras M	2013	16	F	Abdominal pain	None	Patchy hyperemia with erosions	On day 3 of admission	PCR	Intravenous acyclovir followed by oral acyclovir
Sprung B	2016	82	F	Abdominal pain with vomiting, nausea, and anorexia	Chronic lymphocytic leukemia under chemotherapy	Multiple small, hemorrhagic shallow ulcers, and erosions	On day 10 of admission	IHC	NA
Nohr EW	2017	70	F	Abdominal pain with vomiting and nausea	Follicular lymphoma under chemotherapy	Diffuse ulcerative gastritis	Within 24 h of endoscopy	PCR	Intravenous acyclovir followed by oral valacyclovir
Pavlov K	2018	28	F	Thoracic pain and abdominal discomfort	Kidney transplant recipient under immunosuppressive therapy	Mild gastritis with multiple small white lesions	Absent	PCR	Intravenous acyclovir
Matsuo Y	2022	78	M	Abdominal pain	None	Multiple erosions	On Day 3 of admission	PCR	Intravenous acyclovir
Present case		77	M	Abdominal pain with anorexia	Richter syndrome under chemotherapy	Multiple gastric ulcers with surrounding erythema	On Day 3 of admission	IHC	Intravenous acyclovir

*Note:* IHC, immunohistochemistry.

Abbreviations: NA, not available; PCR, polymerase chain reaction; VZV, varicella‐zoster virus.

A review of these cases indicates that patients typically present with nonspecific gastrointestinal symptoms such as abdominal pain, nausea, vomiting, or anorexia. Importantly, cutaneous manifestations often appear after the onset of gastrointestinal symptoms or may be absent at initial presentation [6, 12, 13, 16–18]. This temporal discrepancy contributes to diagnostic delay and underscores the need to consider VZV infection even in the absence of characteristic skin lesions [7, 20, 21].

Endoscopic findings of VZV gastritis are heterogeneous and nonspecific, including erosive gastritis [6, 12, 15, 16], multiple shallow ulcers [14], hemorrhagic lesions [17], or diffuse ulcerative mucosal injury [18]. The endoscopic findings in our patient, consisting of whitish esophageal erosions and multiple gastric ulcers with surrounding erythema, were consistent with previously reported patterns but were insufficiently specific to establish the diagnosis without histopathologic confirmation. Therefore, endoscopic biopsy with virological evaluation plays a crucial role in the diagnostic evaluation of immunocompromised patients with unexplained gastrointestinal lesions.

Histologic diagnosis can be challenging because characteristic viral cytopathic changes, such as intranuclear inclusion bodies and multinucleated giant cells, are not consistently observed. Consequently, virological confirmation using immunohistochemistry or polymerase chain reaction is often required to establish a definitive diagnosis. In the present case, immunohistochemical staining confirmed VZV infection in both esophageal and gastric biopsy specimens, supporting the diagnosis of VZV‐associated esophagogastritis.

Prompt initiation of antiviral therapy is essential in disseminated VZV infection, as delayed treatment has been associated with substantial morbidity and mortality [22]. Intravenous acyclovir remains the standard treatment and has been associated with favorable outcomes when administered early [23–25]. In our patient, initiation of acyclovir therapy led to resolution of both cutaneous and gastrointestinal manifestations.

In addition to early antiviral therapy, prevention of herpes zoster through vaccination is increasingly important, particularly in elderly and immunocompromised populations. The recombinant glycoprotein E–based herpes zoster vaccine has demonstrated high efficacy in preventing herpes zoster and its complications, including in older adults and selected immunocompromised patients [26]. Recent guidelines recommend vaccination in patients with hematologic malignancies and those receiving immunosuppressive therapies when clinically appropriate. Although the present patient had not received zoster vaccination, immunization prior to the initiation of intensive chemotherapy might have reduced the risk of severe viral reactivation. Greater awareness of preventive vaccination strategies may help to decrease the incidence of disseminated and visceral VZV infections in high‐risk populations.

In summary, VZV‐associated esophagogastritis is a rare but clinically significant condition that should be considered in immunocompromised patients presenting with unexplained gastrointestinal symptoms, even before the development of cutaneous lesions. Awareness of this entity and timely endoscopic biopsy with appropriate virological evaluation are essential for early diagnosis and effective treatment.

## Author Contributions

All authors contributed to the study conception and design. Masaya Iwamuro performed data collection and analysis and wrote the first draft of the manuscript. Takehiro Tanaka and Motoyuki Otsuka commented on previous versions of the manuscript.

## Funding

No funding was received for this research.

## Disclosure

All authors read and approved the final manuscript.

## Conflicts of Interest

The authors declare no conflicts of interest.

## Data Availability

The data used to write this case report are available from the corresponding author upon reasonable request.
